# Biological Activity of Copaiba in Damage to the Alveolar Bone in a Model of Periodontitis Induced in Rats

**DOI:** 10.3390/molecules27196255

**Published:** 2022-09-23

**Authors:** Vinicius Ruan Neves dos Santos, João Victor da Silva Motta, Deborah Ribeiro Frazão, Railson de Oliveira Ferreira, Deiweson Souza-Monteiro, Daiane Claydes Baia-da-Silva, Paulo Fernando Santos Mendes, Leonardo Oliveira Bittencourt, João Daniel Mendonça de Moura, Osmar Alves Lameira, Gabriela de Souza Balbinot, Fabrício Mezzomo Collares, Cassiano Kuchenbecker Rösing, Rafael Rodrigues Lima

**Affiliations:** 1 Laboratory of Functional and Structural Biology, Institute of Biological Sciences, Federal University of Pará, Belém 66075-110, PA, Brazil; 2 Laboratory of Biotechnology, Embrapa Amazônia Oriental, Belém 66075-110, PA, Brazil; 3 Dental Materials Laboratory, Faculty of Dentistry, Federal University of Rio Grande do Sul, Porto Alegre 90040-060, RS, Brazil; 4 Department of Periodontology, Faculty of Dentistry, Federal University of Rio Grande do Sul, Porto Alegre 90040-060, RS, Brazil

**Keywords:** alveolar bone loss, Amazonian biodiversity, complementary therapies, copaiba oleoresin, *Copaifera reticulata* Ducke, micro-CT, periodontium, rats

## Abstract

Several studies have investigated the effects of natural products in the treatment of diseases. Traditional Amazonian populations commonly use copaiba due to its well-known anti-inflammatory, antibacterial, and healing properties. In this study, we aimed to investigate the effects of systemic administration of copaiba oleoresin (*Copaifera reticulata* Ducke) on ligature-induced periodontitis in rats. To do so, 21 adult rats were divided into three groups (*n* = 7 each): a control group, ligature-induced periodontitis group, and ligature-induced periodontitis group treated with copaiba oleoresin (200 mg/kg/day). The ligature remained from day 0 to 14, and the copaiba oleoresin was administered via oral gavage during the last seven days. On day 14, the animals were euthanized, and mandibles were collected for histopathological evaluation and microcomputed tomography analysis. Our data showed that the administration of copaiba considerably reduced the inflammatory profile. Moreover, copaiba oleoresin limited alveolar bone loss, increased trabecular thickness and bone-to-tissue volume ratio, and decreased the number of trabeculae compared with those of the untreated experimental periodontitis group. Our findings provide pioneering evidence that supports the potential of copaiba oleoresin in reducing periodontitis-induced alveolar bone damage in rats.

## 1. Introduction

**Figure 1:**
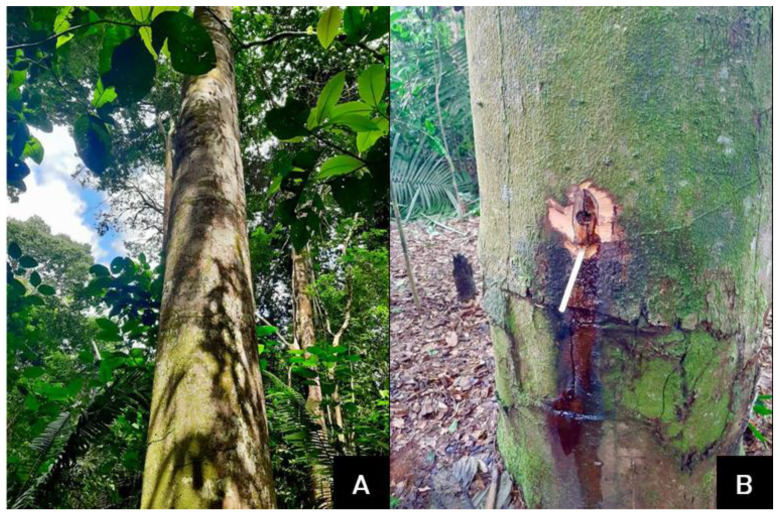
Copaiba tree. (**A**) Trunk of *Copaifera reticulata Ducke*; (**B**) a representative image of extraction of oleoresin by artificial exudation from the trunk.

Based on traditional knowledge, several natural products are used for medicinal purposes, such as copaiba oleoresin obtained from *Copaifera* sp. tree species [1,2] (Figure 1A,B). The copaiba tree belongs to the Fabaceae family, subfamily Caesalpinioideae, and genus *Copaifera* [3]. It is a genus native to Latin America and other tropical regions [3]. There are several species distributed in different parts of the world, and Brazil is the country with the greatest biodiversity of *Copaifera*, with around 60,000 plant varieties [4].

Among the several species of copaiba, one of the most common is the *Copaifera reticulata* *Ducke*, which occurs in the Brazilian Amazon [3]. The oleoresin extracted from the trunk of the *Copaifera reticulata* *Ducke* has compounds such as α-humulene, germacrene, α-copaene, β-elemene, trans-α-bergamotene, β-selinene, α-selinene, β-bisabolene, α-guaiene, trans-β-guaiene, copalic acid, kaurenoic acid, hardwickiic acid, and β-caryophyllene, the last one usually being the main compound [5,6,7,8,9,10,11]. The concentrations of copaiba oleoresin components can be influenced by several factors, including the tree species, soil characteristics, climatic conditions, and the season or period of the year that they are extracted [8,12,13].

Copaiba oleoresin exhibits a variety of biological actions, including anti-inflammatory [2,12,14], antibacterial [15], antifungal [16], and larvicidal [17] activities. Copaiba application in dentistry is a relatively new area of study that is constantly expanding, and most studies have investigated the effects of copaiba on oral pathogens in vitro [11,18,19,20]. A review of the literature indicated the potential of copaiba oleoresin for treating oral pathogens [21].

There has been some animal experimentation involving the effects of copaiba oleoresin, which has shown that it can reduce inflammation and improve healing in the oral mucosa of rats [2,12]. However, there are still no studies on its anti-inflammatory effect in periodontics.

Periodontitis is an inflammatory disease that results from the presence of dysbiotic biofilms on the tooth surface, followed by progressive destruction of the periodontal ligament, and resorption of alveolar bone, which can even lead to tooth loss [22]. Periodontitis treatment may be mechanical (surgical and nonsurgical treatment), with the possibility of using adjunct antimicrobial strategies [23]. Animal models are fundamental in periodontal research to study the relationship between disease and external factors and test potential new treatments. Rats are the animals most commonly used to study the pathogenesis of periodontitis due to similarities in the morphological and developmental aspects of the disease with those of humans, which include gingival area histology and bone and collagen breakdown during alveolar bone loss [24,25].

According to parameters described by Page and Schroeder [26], the onset of periodontitis begins as a response of leukocytes to the metabolic products of the bacteria present in the biofilm, which deregulate homeostasis in the oral environment. This stimulus leads junctional epithelial cells to produce cytokines, such as TNF-α, IL1-β, and IL-6, that trigger a cascade of events that activate innate immunity, culminating in damage to the alveolar bone and other structures of the periodontium [27]. From this perspective, in the search for new adjuvant therapies for the treatment of periodontitis [28], studies have shown the beneficial effects of herbal medicines in the maintenance of alveolar bone integrity in experimental periodontitis models [29,30,31].

Our group has shown in previous studies the effects of copaiba oleoresin on traumatic lesions of the oral mucosa of rats and bioactive effects on human dental pulp stem cells [2,12,32,33], which drove us to investigate the effects on other types of aggravation to the oral cavity, such as periodontitis. Therefore, we aimed to investigate the effects of the systemic administration of copaiba oleoresin on the modulation of the inflammatory response and the architecture of the alveolar bone using a ligature-induced periodontitis model in rats. Our hypothesis was that copaiba oleoresin could modulate the immune response, leading to a protective effect and preventing bone destruction.

## 2. Results

### 2.1. Copaiba Oleoresin Administration Reduced the Intensity of the Inflammatory Response and Preserved Bone Tissue

**Figure 2:**
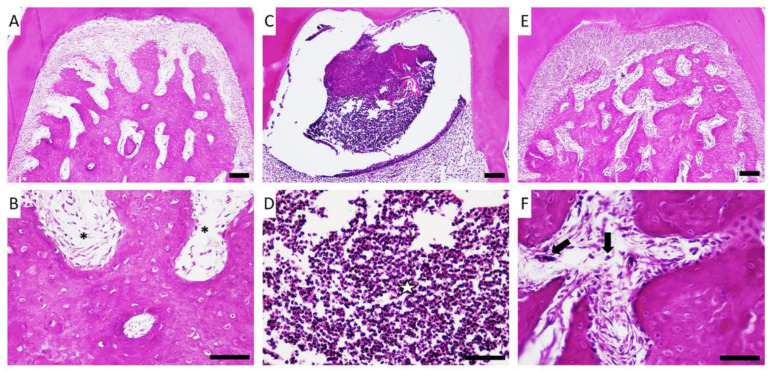
Effects of copaiba oleoresin (200 mg/kg/day for 7 days) in the furcation region of the first molar of animals with experimental periodontitis. Magnifications of 40× and 60× of the furcation region of the mandibular first molar of the control group (**A**,**B**, respectively), experimental periodontitis group (**C**,**D**, respectively), and experimental periodontitis + copaiba oleoresin group (**E**,**F**, respectively). Scale bar = 10 μm. (**B**) Asterisks (*) indicate healthy connective tissue. (**D**) Star (✰) indicates abundant inflammatory tissue. (**F**) Arrows (⬇) indicate inflammatory cells.

When comparing the inflammatory response between the control group (Figure 2A,B) and the group of animals who underwent aggravation without treatment (Figure 2C,D), we detected that applying the ligature for 14 days could cause an intense inflammatory response that was still evident on the 14th day post-lesion, with intense monocytic and lymphocytic infiltrate coupled with a pattern of significant bone loss. When the animals that received copaiba oleoresin treatment were evaluated (Figure 2E,F), the reduced inflammatory infiltration and preservation of the bone trabeculae were notable.

### 2.2. Copaiba Oleoresin Administration Changed the Alveolar Bone Quality and Reduced Alveolar Bone Loss Caused by the Induced Periodontitis Model in Rats

**Figure 3:**
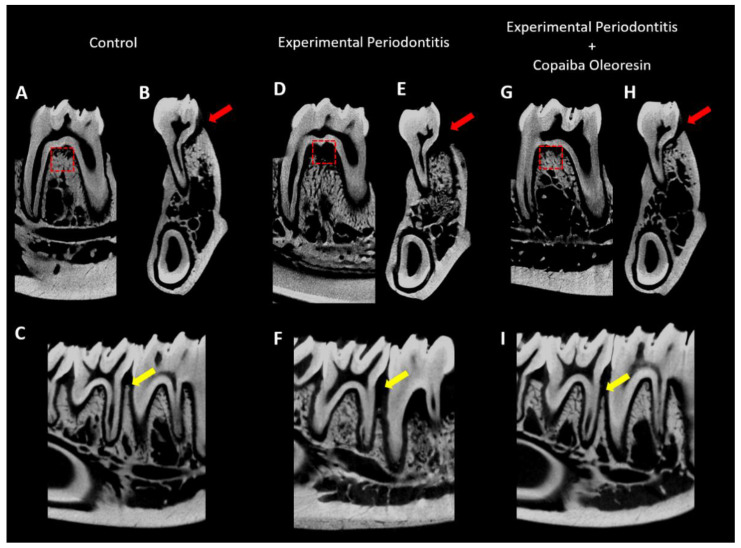
Sagittal (**A**,**C**) and coronal representative slices (**B**) of the animals’ hemimandible in the inferior first molar region of the control group, experimental periodontitis group (**D**,**E**,**F**), and experimental periodontitis + copaiba oleoresin group (**G**,**H**,**I**). Red squares indicate the region of interest for alveolar bone quality evaluation; red arrows highlight the bone loss differences between the groups; yellow arrows highlight the alveolar bone crest level in different groups.

Cortical bone loss was more pronounced in the experimental periodontitis group (Figure 3E) than in the control group (Figure 3B), as shown by the red arrows in Figure 3. However, in the experimental periodontitis group treated with copaiba oleoresin, the cortical bone was significantly preserved (Figure 3H).

Damage to the alveolar bone crest with height reduction was observed in the experimental periodontitis group (Figure 3F) when compared with that of the control group (Figure 3C). In the treated group, preservation of alveolar bone crest height was observed (Figure 3I).

**Figure 4:**
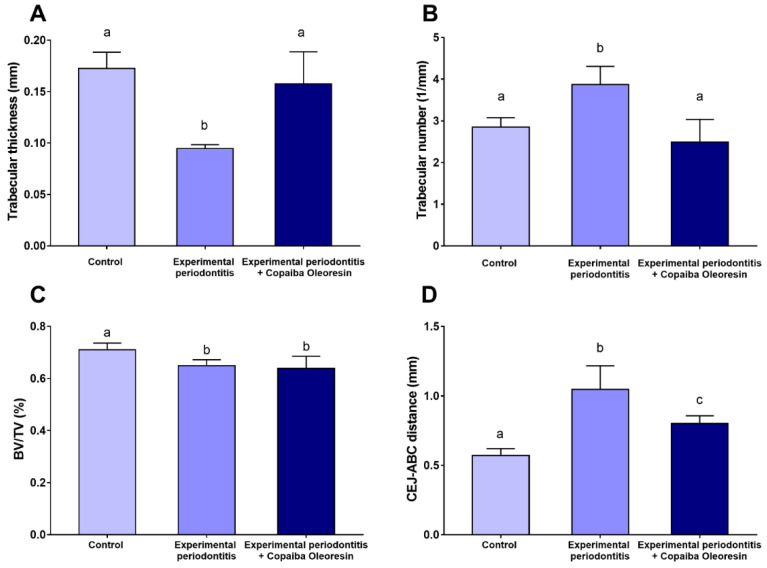
Microcomputed tomography results of the alveolar bone of rats with experimental periodontitis after copaiba oleoresin administration (200 mL/kg/day for 7 days). (**A**) Trabecular thickness (mm), (**B**) trabecular number (1/mm), (**C**) bone-to-tissue volume (BV/TV; %), (**D**) cementum–enamel junction to alveolar bone crest distance (CEJ-ABC; mm). Results are expressed as mean ± SEM. Different letters indicate significant differences between the groups: one-way ANOVA with Tukey’s post hoc test (*p* < 0.05).

Figure 3A,D,G present the region of interest regarding alveolar bone quality. The experimental periodontitis group showed reduced trabecular thickness (Tb.Th) compared with the control group (0.09 ± 0.001 mm vs. 0.17 ± 0.009 mm; *p* = 0.002; Figure 4A) (*n* = 5 for each group). However, experimental periodontitis with copaiba oleoresin administration increased trabecular thickness compared with the periodontitis group (0.15 ± 0.01 mm vs. 0.09 ± 0.001 mm; *p* = 0.008; Figure 4A). Experimental periodontitis increased the number of trabeculae (Tb.N) compared with the control group and with the experimental periodontitis with copaiba oleoresin administration group (3.86 ± 0.21 1/mm vs. 2.85 ± 0.13 1/mm vs. 2.49 ± 0.27 1/mm; *p* < 0.05; Figure 4B) (*n* = 5 for each group). The groups subjected to experimental periodontitis showed a reduction in the bone-to-tissue volume ratio compared with the control group (0.71 ± 0.01% vs. 0.64 ± 0.01% vs. 0.63 ± 0.02%; *p* < 0.05) (*n* = 5 for each group) (Figure 4C).

**Figure 5:**
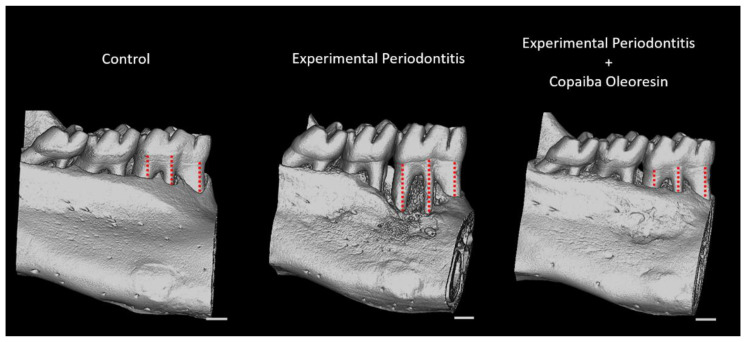
Representative images of the three-dimensional reconstructions of the hemimandible in the control, experimental periodontitis, and experimental periodontitis with copaiba oleoresin administration groups. Red dotted lines indicate the vertical levels analyzed to measure alveolar bone loss. Scale bar = 1 mm.

These findings support the results of the 3D assessment of alveolar bone loss (Figure 5). Compared with the experimental periodontitis group, copaiba oleoresin administration preserved the vertical dimensions of the alveolar bone (1.04 ± 0.08 mm vs. 0.80 ± 0.02 mm; *p* = 0.01; Figure 4D) (*n* = 5 for each group), as illustrated in Figure 5.

## 3. Discussion

In this study, we investigated the use of copaiba oleoresin on the modulation of inflammation and preservation of alveolar bone structure after injury by ligature-induced experimental periodontitis. The results indicated a beneficial effect of exposure to copaiba oleoresin on the occurrence of alveolar bone loss in rats. Mechanisms linked to such results need to be understood. The histopathology evaluation showed an attenuation of the local inflammatory process in experimental periodontitis with copaiba oleoresin administration. In addition, the copaiba oleoresin prevented a reduction in trabecular thickness and reduced the loss of vertical dimensions of the alveolar bone caused by the injury. These findings suggest a potential protective action of copaiba oleoresin against damage caused by periodontitis to the alveolar bone structure. As periodontitis represents an inflammatory condition triggered by dysbiotic biofilms, the inflammatory axis is important for pathogenesis [27]. In this sense, the modulation of the inflammatory process, as demonstrated in the present study, is of interest.

The model of injury to the alveolar bone in rats mimics the processes that occur during periodontitis in humans [25]. The insertion of a silk or cotton thread around the first molar forms a retentive area of biofilm in which bacteria involved in the pathogenesis of periodontal disease, such as *Porphyromonas gingivalis*, *Prevotella nigrescens,* and *Aggregatibacter actinomycetemcomitans*, may be present [27]. Due to the parallels with the pathophysiology of adult human periodontitis, the ligature-induced periodontitis model in rodents is viable and the most-used method in investigations due to its ability to generate alveolar bone loss through an inflammatory condition [25,34]. In addition, animal studies are of utmost importance in increasing our understanding of the mechanisms involved in the pathophysiology of periodontal disease. Vargas-Sanchez et al., in 2017, validated the experimental periodontitis induction protocol, which effectively induced alveolar bone loss during the same experimental period used in this study [25]. In this context, our choice of a single period of periodontitis analysis of 14 days is valid because it is a period during which significant bone loss can already be detected, and this could be seen in our animals. Additionally, it was demonstrated that after such a period, periodontal breakdown may not present significant progression [25,35,36]. Consequently, this timeframe could provide evidence about whether copaiba oleoresin is able to reduce the damage.

The oleoresin of *Copaifera reticulata* has several compounds that guarantee its various biological activities, and these are mainly attributed to its main compounds [2,3,5,12,14,15,37]. In this study, when *Copaifera reticulata* *Ducke* was characterized by gas chromatography mass spectrometry (GC-MS) [10], it presented β-caryophyllene as a major component (37.3%), which is a ligand of the cannabinoid receptor 2 [37]. When binding to this receptor, it blocks the activation of NF-κB, decreasing the synthesis of interleukin 6 (IL-6) [37,38]. This compound also downregulates other proinflammatory cytokines, such as IL-1β, prostaglandin E2, and tumor necrosis factor-alpha, as well as anti-inflammatory cytokines such as IL-6 [14,37,39,40]. Additionally, some investigations have shown that β-caryophyllene can minimize the expression of nitric oxide (NO), which is a free radical associated with periodontitis, because it is related to an inflammatory process and can induce osteoclast activation [13,41,42]. Therefore, β-caryophyllene may have been able to modulate the inflammatory response caused by ligature periodontitis, reducing bone damage. As a result, our histopathological findings showed that copaiba oleoresin therapy significantly reduced inflammation, leaving a highly reduced number of inflammatory cells. Conversely, the untreated experimental periodontitis group presented abundant inflammatory patterns. Consequently, considering that an inflammatory process may lead to morphological alterations, micro-CT analyses were performed to visualize the role of copaiba oleoresin in mediating the interactions between inflammation and alveolar bone structure.

In this context, micro-CT is considered the gold standard for assessing bone microarchitecture [43,44,45]. This analysis enables high-resolution three-dimensional reconstruction of bone, which provides accurate micrometer-to-submicrometer structural determination of various parameters [46]. Here, the vertical bone loss parameter allowed us to evaluate the primary structural damage to the alveolar bone caused by the experimental periodontitis model, associating it with the protection provided by copaiba oleoresin [45,46]. Furthermore, the investigation of bone quality through the analysis of Tb.Th, Tb.N, and BV/TV indicated the changes in alveolar bone microarchitecture generated by the induction of periodontitis and the administration of copaiba oleoresin [43,44]. The results of micro-CT evaluation showed that the copaiba oleoresin prevented alveolar bone loss and preserved bone quality compared to the experimental periodontitis group. The loss of trabecular thickness of the alveolar bone, generated by experimental periodontitis, was mitigated by administering copaiba oleoresin.

The mechanisms of alveolar bone loss in periodontitis involve the complex innate and acquired immune responses and are also associated with the role of periodontal pathogens’ lipopolysaccharides (LPS) in the activation of osteoclastogenesis [47]. PGE2, IL1-β (generated by TH1-helper lymphocytes), TNF-α, and IL-6 are released when LPS interacts with Toll-like receptors (primarily TLR-2, TLR-6, and TLR-9) on PMNs, dendritic cells, lymphocytes (TH1-helper lymphocytes), epithelial cells, and fibroblasts [48]. *Porphyromonas gingivalis, Escherichia coli, Tannerella forsythia, Prevotella intermedia, Prevotella nigrescens,* and *Treponema denticola* lipopolysaccharides are associated with TLR-2 and TLR-9. TLR-4 and TLR-4 are associated with the lipopolysaccharides of *E. coli*, *Aggregatibacter actinomycetemcomitans,* and *Veillonella parvula* [27]. Those interactions and released cytokines are part of a complex network of interactions that lead osteoblasts to produce RANK [26]. A reduction in the production of its antagonist osteoprotegerin by osteoblasts and the differentiation of myeloid progenitors from osteoclasts are noted when RANKL binds to RANK receptors [27]. In this way, the Toll-like receptor and inflammation-induced osteoclastogenesis pathway are linked to bone loss [49]. Even when using the ligature-induced periodontitis model, authors indicated that a biofilm containing *Porphyromonas gingivalis, Aggregatibacter actinomycetemcomitans,* and *Tannerella forsythia* is present in the periodontal tissue of rats [35] and causes visual bone loss within 3 to 7 days [25].

Studies have demonstrated that copaiba oleoresin shows antibacterial properties against pathogens, such as *Fusobacterium nucleatum, Streptococcus mitis* [11], *Aggregatibacter actinomycetemcomitans,* and *P. gingivalis* strains [50]. These periodontal pathogens have LPS that activate osteoclasts, promoting alveolar bone loss in periodontitis [47]. The bactericidal effects on *Fusobacterium nucleatum* may be associated with a reduction in the pathogenesis of periodontitis [47]. Additionally, polyalthic acid, kaurenoic acid, and hardwickiic acid, components of copaiba oleoresin, showed bactericidal and bacteriostatic effects on *Aggregatibacter actinomycetemcomitans* and *P. gingivalis* strains [50]. Although our findings do not include a microbiological analysis, the findings in the literature allowed us to hypothesize that not only the anti-inflammatory but also the antimicrobial action of copaiba may have been closely linked to the mitigation of alveolar bone loss in this study.

Thus, we must recognize that dental biofilm, with its intricacy of interactions between diverse microorganisms, is the primary etiological element in periodontitis [22]. The risk of a microbiological imbalance diminished due to the bactericidal activity of copaiba oleoresin on *F. nucleatum*, which may be associated with reducing harmful effects on periodontal tissues, such as alveolar bone loss [11]. However, the effects of copaiba on planktonic bacteria should differ in biofilm owing to its complexity [20]. Thus, further studies may consider evaluating the antibiofilm effects of copaiba oleoresin to confirm bacteriostatic and bactericidal effects in periodontal tissue surrounded by dental biofilm, as well as investigating the possible mechanisms of action in cytokines.

## 4. Materials and Methods

### 4.1. Animals and Experimental Groups

This study was approved by the Ethical Committee for the Use of Animals of the Federal University of Pará (UFPA) (No. 2478020320). Twenty-one male *Rattus norvegicus* (Wistar) rats, 60 days old, weighing 150–200 g, were provided by the Federal University of Pará central animal room and randomly divided into three experimental groups (*n* = 7 per group): a control group, an experimental periodontitis group, and an experimental periodontitis group treated with copaiba oleoresin. Animals were maintained under a 12 h light/dark cycle at a controlled temperature (25 ± 1 °C), and received water and food ad libitum.

### 4.2. Plant Material, Characterization, and Acute Oral Toxicity Test

**Figure 6:**
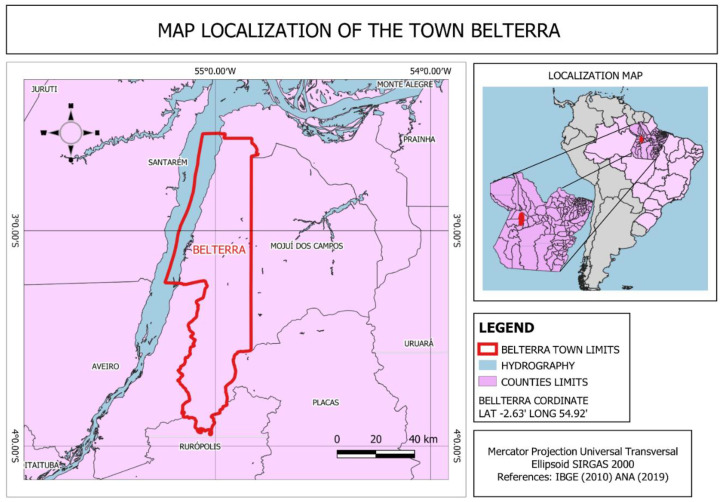
The geographical location of the town Belterra in the state of Pará, Brazil.

Oleoresin acquisition followed the same protocol described in previous studies [10,12]. It was collected and characterized by researchers at the Eastern Amazon Agroforestry Research Center, EMBRAPA, Eastern Amazon. Oleoresin was extracted by artificial exudation from the trunk of *Copaifera reticulata* *Ducke*; specifically, an approximately 30-year-old native tree in the city of Belterra, Pará state, Brazil (Latitude: 02°38”11” S, Longitude: 54°56”14” W) (Figure 6). After collection, the oleoresin was stored away from light, oxygen, and heat to stabilize its volatile compounds. Specimens of this plant were deposited in the IAN EMBRAPA Herbarium (Exsiccate: 183,939). Characterization of samples for gas chromatography mass spectrometry (GC-MS) was described by Guimarães-Santos et al. in 2012, and presented β-caryophyllene (37.3%), β-bisabolene (14.5%), and trans-α-bergamotene (9.0%) as major components of copaiba oleoresin [10].

The copaiba oleoresin dose was determined in accordance with Teixeira et al., who performed the determination based on OECD Toxicity guideline tests in 2017 [12]. The dose of 200 mg/kg/day was prepared as an emulsion, using 5% tween 20 + saline solution as surfactant and aqueous phase [12,33].

### 4.3. Induction of Experimental Periodontitis and Administration of Copaiba Oleoresin

On the first day of the experiment, all animals were anesthetized with xylazine hydrochloride (8 mg/kg) (Syntec do Brasil LTDA, São Paulo, Brazil) and ketamine hydrochloride (75 mg/kg) (Ceva do Brasil LTDA, São Paulo, Brazil). A cotton thread dressing (Coats, Corrente, São Paulo, Brazil) was placed around the cervical region of the mandibular first molars to induce periodontitis and maintained until euthanasia (14 days) [25]. Animals in the copaiba oleoresin group received 200 mg/kg/day of copaiba oleoresin by gavage from day 7 until 14 of the experimental period. Control animals were only gavaged with distilled water without ligature-induced experimental periodontitis. For bodyweight measurement, all animals were weighed weekly.

### 4.4. Sample Collection

**Figure 7:**
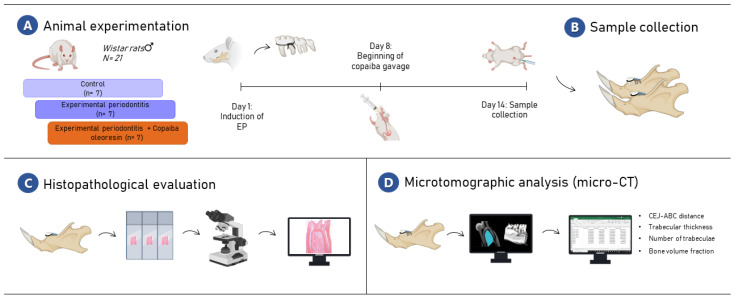
The methodological figure of the experimental steps. In (**A**), the experimental stages of the study are displayed; In (**B**), the sample collection stage is represented; In (**C**), the histopathological evaluation is represented by the light field microscopy schematic figure; In (**D**), the microtomographic analysis (micro-CT) is displayed. This figure was created with BioRender.com, accessed on 25 July 2022.

After the experimental period, the animals were intraperitoneally anesthetized with xylazine hydrochloride (30 mg/kg) and ketamine hydrochloride (180 mg/kg) to be perfused with 0.9% saline, heparinized, and followed by 4% formaldehyde administered through the heart left ventricle. Then, one hemimandible was post-fixed in 4% formaldehyde in a liquid volume at least 30 times larger than the piece for microcomputed tomography analysis. The other hemimandibles were post-fixed in 10% formaldehyde solution for 24 h and kept for 90 days in 10% ethylenediaminetetraacetic acid solution until processing for histological analysis. The sample description and methodological steps are summarized in Figure 7.

### 4.5. Gross Histopathological Evaluation

Following demineralization, the pieces were dehydrated in alcohol, diaphanized in xylene, and embedded in paraplast. After inclusion, the materials were sliced with a Leica RM 2045 microtome (Leica Microsystems, Nussloch, Germany) in the vestibule-lingual direction with a thickness of 5 µm and placed on separate slides. Sections were stained with hematoxylin and eosin and photomicrographed using a digital color camera (Sony Cyber-Shot DSC W-230, 4× optical zoom, Tokyo, Japan) connected to an optical microscope (Leica QWin Pl–s-Leica Microsystems, Nussloch, Germany). The periodontitis inflammatory profile was determined in semiserial sections across the mandible length. The severity of the inflammation was defined by the intensity, characteristics, and extent of the inflammatory infiltration and the bone integrity.

### 4.6. Microcomputed Tomography (Micro-CT) Analysis

Micro-CT scans of the animals hemimandibles were performed (MicroCT.SMX-90 CT; Shimadzu Corp., Kyoto, Japan). Images were obtained with a 360° rotation and an intensity of 70 kV and 100 mA. Then, they were reconstructed using inspeXio SMX-90CT software (Shimadzu Corp., Kyoto, Japan), which produced 541 images per sample with a voxel size of 10 µm and a resolution of 1024 × 1024.

RadiAnt DICOM Viewer 5.0.1 (Medixant, Poznan, Poland) was used to assess the three-dimensional (3D) reconstruction of the hemimandibles. The 3D models were positioned in standard orientations, allowing the buccal and lingual tooth faces to be observed. Thus, alveolar bone vertical levels were evaluated by measuring the distance between the cementum–enamel junction (CEJ) and the alveolar bone crest (ABC) at six selected points of the first inferior molar (i.e., mesiobuccal, buccal, distobuccal, distolingual, lingual, and mesiolingual), and then averaged [34].

ImageJ^®^ (National Institutes of Health, Bethesda, MD, USA) software was used on 70 slices of the inferior first molar alveolar bone region to evaluate the quality of the alveolar bone tissue. The inter-radicular zone, near the furcation area, was chosen as the region of interest (average size of 0.200 mm^2^). The threshold was set at 110–240 to binarize the different gray colors. The trabecular thickness (Tb.Th), trabecular number (Tb.N), and percentage of bone volume to tissue volume (BV/TV) were calculated using the BoneJ plug-in (National Institutes of Health, Bethesda, MD, USA).

### 4.7. Statistical Analyses

To test the normality of the data, the statistical Shapiro–Wilk test was performed. One-way ANOVA was then applied, followed by Tukey’s post-test for comparison between groups, allowing a statistical significance level of *p* < 0.05. GraphPad Prism 8.0.2 software (San Diego, CA, USA) was used for all analyses. The data are expressed as the mean ± standard error of the mean (SEM).

## 5. Conclusions

Copaiba oleoresin was able to reduce the inflammatory process generated by - periodontitis. In addition, oleoresin administration resulted in less alveolar bone loss even in the presence of periodontitis. Our results provided initial evidence that corroborates the applicability of copaiba oleoresin in mitigating the alveolar bone damage caused by periodontitis. Therefore, it is essential to carry out more in-depth research on the effects of copaiba oleoresin on the modulation of periodontal diseases in order to answer the new questions raised by this study.

## Data Availability

All data are available in the article.

## Sample Availability

Samples of the compounds are available from the authors.
